# Preparation of hierarchical mesoporous CaCO_3_ by a facile binary solvent approach as anticancer drug carrier for etoposide

**DOI:** 10.1186/1556-276X-8-321

**Published:** 2013-07-15

**Authors:** Haibao Peng, Kun Li, Ting Wang, Jin Wang, Jiao Wang, Rongrong Zhu, Dongmei Sun, Shilong Wang

**Affiliations:** 1 School of Life Science and Technology, Tongji University, 1239 Siping Road, Shanghai 200092, People's Republic of China

**Keywords:** CaCO_3_ nano-structure, Multistage self-assembled, pH-sensitive drug delivery, Etoposide

## Abstract

To develop a nontoxic system for targeting therapy, a new highly ordered hierarchical mesoporous calcium carbonate nanospheres (CCNSs) as small drug carriers has been synthesized by a mild and facile binary solvent approach under the normal temperature and pressure. The hierarchical structure by multistage self-assembled strategy was confirmed by TEM and SEM, and a possible formation process was proposed. Due to the large fraction of voids inside the nanospheres which provides space for physical absorption, the CCNSs can stably encapsulate the anticancer drug etoposide with the drug loading efficiency as high as 39.7 wt.%, and etoposide-loaded CCNS (ECCNS) nanoparticles can dispersed well in the cell culture. Besides, the drug release behavior investigated at three different pH values showed that the release of etoposide from CCNSs was pH-sensitive. MTT assay showed that compared with free etoposide, ECCNSs exhibited a higher cell inhibition ratio against SGC-7901 cells and also decreased the toxicity of etoposide to HEK 293 T cells. The CLSM image showed that ECCNSs exhibited a high efficiency of intracellular delivery, especially in nuclear invasion. The apoptosis test revealed that etoposide entrapped in CCNSs could enhance the delivery efficiencies of drug to achieve an improved inhibition effect on cell growth. These results clearly implied that the CCNSs are a promising drug delivery system for etoposide in cancer therapy.

## Background

Because of drug resistance, low bioavailability, and undesired severe side effects, the therapeutic effect of chemotherapy has been greatly limited for the treatment of cancer [1-5]. The application of many antitumor drugs, such as curcumin, PPT, and etoposide, has been limited because of their disadvantages of poor water solubility, metabolic inactivation, myelosuppression, side effect for normal tissue, and poor targeting [6].

To overcome these limitations, drug delivery techniques have been intensively investigated and studied to improve the therapeutic effect [7]. Compared with conventional formulations, an ideal anticancer drug delivery system shows numerous advantages compared with conventional formulation, such as improved efficacy, reduced toxicity, and reduced frequency of doses [8]. Besides, the nanocarriers for anticancer drugs can also take advantage of the enhanced permeation and retention (EPR) effect [9-11] in the vicinity of tumor tissues to facilitate the internalization of drugs in tumors. Drug carriers with diameters less than 600 nm may be taken up selectively by tumor tissues because of the higher permeation of tumor vasculature [12]. Multiplicity carrier and functional nanoparticles exhibit greatly enhanced therapeutic effects and can improve the dispersion stability of the particles in water and endow the particles with long circulation property *in vivo*[8,12-18]. However, the nanoscale drug delivery systems may also exhibit some disadvantages, such as poor biocompatibility, incompletely release *in vivo*, and incomplete degradation. Therefore, people are constantly developing delivery systems which are easily prepared, environment-friendly, and biocompatible.

CaCO_3_, the most common inorganic material of the nature, widely exists in living creatures and even in some human tissues. There are a large number of reports on calcium carbonate in recent years, but not so much attention has been focused on its biological effects. Compared with other inorganic materials, CaCO_3_ has shown promising potential for the development of smart carriers for anticancer drugs [19] because of its ideal biocompatibility, biodegradability, and pH-sensitive properties, which enable CaCO_3_ to be used for controlled degradability both *in vitro* and *in vivo*[20]. It has been used as a vector to deliver genes, peptide, proteins, and drug [21-23]. Furthermore, spherical CaCO_3_ particle might be found in its uses in catalysis, filler, separations technology, coatings, pharmaceuticals and agrochemicals [24,25].

Etoposide, a derivative of the anticancer drug podophyllotoxin, is an important chemotherapeutic agent for the treatment of cell lung cancer [26], testicular carcinoma [27], and lymphomas [28]. Its direct applications had been limited by its poor water solubility, side effect for normal tissue, and poor targeting. Therefore, an efficient drug delivery system is desired to overcome these drawbacks and improve its clinical therapy efficiency.

Considering that the dimensional and structural characteristics of the materials endow them a wide range of remarkable properties and potential applications, the design and synthesis of materials with specific morphologies have drawn significant attention [29-31]. Among the different morphological nanostructures, the hierarchical particles from nanometer to micrometer dimensions reveal the great desirable properties. They have been attracting considerable attention, owing to their widespread applications in catalysis, chemical reactors, drug delivery, controlled release of various substances, protection of environmentally sensitive biological molecules, and lightweight filler materials [19,32-37]. Highly orderly hierarchical and pH value-sensitive calcium carbonate can stably preserve drug under physiological conditions and selectively release in the intracellular acid environment [38]. Han et al. reported the mesoporous hollow CaCO_3_ spheres prepared in guanidinium ionic liquid, but the surface area of those products is very low, even only 17 m^2^/g [39]. It is still attractive to prepare mesoporous high-surface area carbonates with unique morphology and structure.

**Figure 1:**
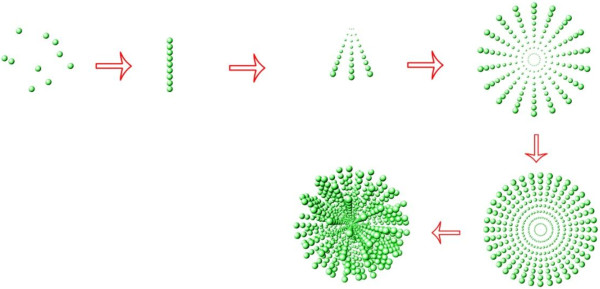
Schematic illustration for the synthesis of CCNSs.

Herein, a crystallization of mesoporous calcium carbonate nanospheres (CCNSs) with hierarchical structure was prepared by a new facile binary solvent method which is involved in the multistage self-assembly of calcium carbonate crystallites into hierarchical spheres under the templating effect of CO_2_ (as shown in Figure 1). These prepared CCNSs have high surface areas, even up to 82.14 m^2^/g, and show the typical mesoporous properties. The method is mild, easily performed, and environment-friendly, which is based on a biomimetic system supported liquid membrane used by Sun [40] and mixed-solvent method used by Qian [41]. Etoposide-loaded strontium carbonate nanoparticles have been studied by our group [41]. However, there could be an existing problem about the enrichment of strontium toxicity after strontium carbonate degradation *in vivo*. Therefore, CCNSs were used as the carrier for etoposide in this study; the drug loading efficiency and the drug release behaviors were also evaluated. Moreover, *in vitro* cellular experiments with MTT (3-[4,5-dimethylthiazol-2-yl]-2,5-diphenyl tetrazolium bromide) assay and fluorescence-activated cell sorter (FACS) analysis were carried out to evaluate the anticancer effect of etoposide-loaded CCNSs. Meanwhile, confocal laser scanning microscopy (CLSM) image was utilized to investigate the uptake of CCNSs by cancer cells. The possible mechanism of the targeted delivery of the ECCNSs was also discussed based on the obtained results and related references.

## Methods

### Materials

Etoposide (≥98%) was a kind gift from the University of Science and Technology of China. Dimethyl sulfoxide (DMSO) and MTT formazan were purchased from Sigma Chemical Co. (St Louis, MO, USA). CaCl_2_ (analytical reagent (AR)), Na_2_CO_3_ (AR), citric acid (AR), HCl (36%–38%), and ethanol (AR) were purchased from Sinopharm Chemical Regent Co., Ltd. (Shanghai, China) and were used without further purification. Dulbecco's modified Eagle medium (DMEM) (high glucose), RPMI-1640, fetal calf serum (FCS), penicillin G, streptomycin, and trypsinase were obtained from GIBCOBRL (Grand Island, New York, NY, USA). Deionized water was decarbonated by boiling before its use in all of the applications.

#### Synthesis of etoposide-loaded calcium carbonate nanospheres

All the experiments were prepared at room temperature. Etoposide-loaded calcium carbonate nanospheres were synthesized by mixing calcium chloride and sodium carbonate aqueous solution in the presence of ethanol, citric acid, and etoposide. Etoposide (0.2 g) and 10 mL CaCl_2_ (0.1 M) were dissolved in 60 mL mixed solvent of ethanol and deionized water (volume ratio = 1:2), marked as solution A. Na_2_CO_3_ (0.02 g) and 10 mL of Na_2_CO_3_ (0.1 M) were dissolved in 60 mL mixed solvent of ethanol and deionized water (volume ratio = 1:2), marked as solution B. Solution B was added dropwise to the vigorously stirred solution A. With the reaction proceeding, the milky white precipitation was obtained after 72 h at room temperature. The precipitation was washed thrice with mixed solvent of ethanol and deionized water (volume ratio = 1:2) and dried using vacuum freeze drier. The blank carrier CCNSs were prepared without the addition of etoposide, and other experimental parameters were similar to the ECCNSs sample.

#### Characterization

The morphology of the ECCNSs was viewed by field-emission scanning electron microscopy (Hitachi S4800, Chiyoda-ku, Japan) at an acceleration voltage of 1 to 5 kV and a JEOL 1230 transmission electron micrograph (TEM, Akishima-shi, Japan) at an acceleration voltage of 200 kV. Brunauer-Emmett-Teller (BET) surface area and pore distribution of the CaCO_3_ products were determined from N_2_ adsorption-desorption isotherms using a Micromeritics TriStar 3000 system (Norcross, GA, USA). The zeta potential distribution of nanoparticles was analyzed by Nano ZS, Malvern Instruments Ltd., Southborough, MA, USA. Fourier transform infrared measurement was recorded on a Bruker Vector 22 spectrophotometer (Madison, WI, USA) in the range of 4,000 to 500 cm^−1^ using the standard KBr disk method (sample/KBr = 1/100). UV–vis spectra were measured on a CARY50 spectrophotometer (Varian, Victoria, Australia). The crystallographic structure of the solid samples was recorded using an X-ray diffraction (XRD, Bruker D8) with Cu Kα radiation (*λ* = 0.154056 nm) (Karlsruhe, Germany), using a voltage of 40 kV, a current of 40 mA, and a scanning rate of 0.02°/s, in 2*θ* ranges from 10° to 70°. The average particle size (z-average size) and size distribution were measured by photon correlation spectroscopy (LS230 Beckman Coulter, Fullerton, CA, USA) under a fixed angle of 90° in disposable polystyrene cuvettes at 25°C.

#### Sedimentation study in RPMI-1640 medium

Etoposide (5 mg) was placed in a centrifugal tube of 15 mL and resuspended with 10 mL RPMI-1640 medium supplemented with 10% fetal bovine serum and 1% penicillin-streptomycin solution. The tube then stood for over 4 h after vortexing for 2 min. Photographs of the sample were taken at 1 h, 2 h, and 4 h. Other samples were studied in the same way. Based on the drug encapsulation efficiency, the same quantity of etoposide was applied to all formulations for the sedimentation study.

#### Determination of loading amount and *in vitro* release test

The amount of incorporated etoposide was measured through UV–vis spectroscopy. A known weight of ECCNS sample was placed in a 10-mL flask, then 100 μL of 3 M HCl solution was subsequently added into it, and the flask was filled with 100% phosphate buffer solution (PBS) (pH = 7.4) until total volume reached 10 mL. After the ECCNS sample was totally dissolved, the concentration of etoposide was determined with a UV–vis spectrophotometer at 285 nm. The concentration of etoposide was calculated according to an already obtained calibrating curve of etoposide (Abs = 0.00645c + 0.01599, *r* = 0.99923). The drug loading capacity and encapsulation efficiency are calculated as follows:

$$\begin{array}{ll} \mathrm{drug}\;\mathrm{loading}\;\mathrm{capacity}= & \frac{\mathrm{weight}\;\mathrm{of}\;\mathrm{etoposide}\;\mathrm{in}\;\mathrm{ECCNSs}}{\mathrm{weight}\;\mathrm{of}\;\mathrm{ECCNSs}}\\ & \times 100\% \end{array}$$

The etoposide release test was performed in 180 mL PBS at pH 7.4, 5.8, and 3.0. ECCNS (25 mg) was resuspended in 10 mL PBS and loaded in a dialysis bag. The release system was swayed in a bath reciprocal shaker at 100 rpm and at constant temperature of 37°C above for 120 h. Aliquots (2 mL) were extracted at desired time intervals, and another 2 mL fresh PBS was added to the system. The accumulated amount of etoposide released was determined by UV absorption at 285 nm.

#### Cytotoxicity assay

Cytotoxicity was characterized by MTT test through the human embryonic kidney (HEK) 293 T cells. 293 T cells with a density of 1 × 10^4^ cells/well were seeded on a 96-well polystyrene plate, and each well contained 100 μL of DMEM (high glucose) medium supplemented with 10% fetal bovine serum and 1% penicillin-streptomycin solution. Cells were subsequently incubated at 37°C in a 5% CO_2_ humid incubator for 24 h. CCNSs, etoposide, and ECCNSs were added to the wells with concentrations 5, 10, 20, and 40 μg/mL in sequence. The HEK 293 T cells were incubated as described above for 24 and 48 h. A control experiment was performed with pure culture medium without treatment. Then, 20 μL (5 mg/mL) of MTT was added to each well, and the plate was further incubated for 4 h to deoxidize MTT under light-blocking condition. After removal of the MTT dye solution, cells were treated with 150 μL DMSO, and the absorbance at 490 nm was measured using ELX 800 UV reader (BioTek, Winooski, VT, USA), and cell viability was calculated by:

$$\begin{array}{ll} \mathrm{Cell}\;\mathrm{viability}\;\left(\%\right)= & \frac{{\mathrm{OD}}_{490}\left(\mathrm{test}\right)-{\mathrm{OD}}_{490}\left(\mathrm{blank}\right)}{{\mathrm{OD}}_{490}\left(\mathrm{control}\right)-{\mathrm{OD}}_{490}\left(\mathrm{blank}\right)}\\ & \times 100\% \end{array}$$

#### Inhibition against SGC-7901 cells

The antitumor effect of CCNSs, etoposide, and ECCNSs against human gastric carcinoma (SGC-7901) cells was examined by cell viability test. SGC-7901 cells with a density of 8 × 10^4^ cells/well were seeded on a 96-well polystyrene plate, and each well contained 100 μL of RPMI-1640 medium supplemented with 10% fetal bovine serum and 1% penicillin-streptomycin solution. Cells were incubated at 37°C in a 5% CO_2_ humid incubator for 24 h. Triplicate wells were treated with CCNSs, free etoposide, and ECCNSs in different concentrations of 5, 10, 20, and 40 μg/mL. These SGC-7901 cells were incubated as described above for 24 and 48 h. MTT of 20 μL (5 mg/mL) was added to each well before the cells were incubated for 4 h at 37°C under light-blocking condition. After the removal of the MTT dye solution, cells were treated with 150 μL DMSO. Absorbance was measured at 490 nm using ELX 800 reader, and inhibition against SGC-7901 cells was calculated by the following equation:

$$\begin{array}{ll} \mathrm{Inhibition}\;\left(\%\right)= & \left[1-\frac{{\mathrm{OD}}_{490}\left(\mathrm{test}\right)-{\mathrm{OD}}_{490}\left(\mathrm{blank}\right)}{{\mathrm{OD}}_{490}\left(\mathrm{control}\right)-{\mathrm{OD}}_{490}\left(\mathrm{blank}\right)}\right]\\ & \times 100\% \end{array}$$

#### Fluorescence activated cell sorter analysis

The number of the apoptosis cells was determined with the Annexin V-PI detection kit (KeyGEN Biotech). SGC-7901 cells with 1 × 10^6^ were cultured, suspended in RPMI-1640 with 10% pasteurized FCS, and seeded on a 24-well flat-bottomed plate and incubated for 24 h at 37°C. The free etoposide, ECCNSs, and culture medium were only added to each group with the concentration of 30 μg/mL. Based on the drug encapsulation efficiency, the same quantity of etoposide was applied to all formulations for the apoptosis analysis. The incubation continued for 24 h at 37°C. Then, the cells were harvested and washed with PBS, and then PI and Annexin V were added directly to the cell suspended in the binding buffer (10 mM HEPES, 140 mM NaCl, 2.5 mM CaCl_2_, pH 7.4). The cells were incubated in the dark for 15 min at 37°C and submitted to FACS analysis on a Beckton-Dickinson (Mountain View, CA, USA) spectrophotometer.

#### Confocal laser scanning microscopy

CLSM images of the ECCNSs and etoposide were obtained using confocal laser scanning microscope (Leica, Wetzlar, Germany) equipped with an oil immersion objective (60×, Zeiss, Oberkochen, Germany). A suspension of the particles was placed on a glass slide and dried prior to use. Fluorescence images were obtained at an excitation wavelength of 488 nm (fluorescein isothiocyanate (FITC)) and 405 nm (4',6-diamidino-2-phenylindole (DAPI)).

## Results and discussion

**Figure 2:**
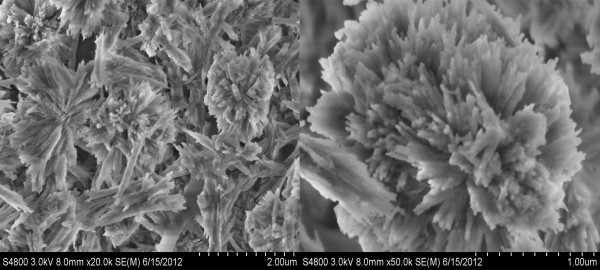
SEM images of ECCNSs.

As shown in Figure 1, CCNSs were obtained by a multistage self-assembled strategy. In this study, a series of intermediates were trapped, in order to confirm the formation process of the CCNSs. It was found that the nanoparticles firstly concentrated and arranged in a line at an early stage. Then, the particles grew rapidly into the broom shape via crystallization of nanoparticles coupled with a simultaneous multiscale assembly. With the reaction going on, the broom-like structure formed into a high-order spherical structure, as shown in Figure 2. The CCNSs were synthesized by a binary solvent method. Firstly, the reaction of citric acid with HCO_3_^−^ ions generates CO_2_ bubbles and H_2_O. And then, the CO_2_ bubbles serve as not only the template of engineered nanospheres but also the reactive materials (reaction formulas listed below). Furthermore, citric acid acts as a crystal modifier to control the selectivity of polymorph and crystal morphology. Citric acid may be coupled by hydrogen bond with etoposide, which can be suggested from the structural formula. Then, the carbanion of citric acid can bond with calcium ions. Accordingly, the synthesis of ECCNSs based on high concentration of hydrophobic drugs (etoposide) could be involved in the crystallization of CCNSs in alcohol-water systems where alcohol can be volatile slowly during the rapid stirring synthetic system. As the ions in blood can destroy hydrogen bonds, the drug will be released from the synthetic calcium carbonate nanospheres.

$$\begin{array}{c} 2\mathrm{RCOOH}\left(\mathrm{aq}\right)+{\mathrm{CO}}_{3}^{2-}=2{\mathrm{RCOO}}^{-}+H_{2}O\left(\mathrm{aq}\right)+{\mathrm{CO}}_{2}\left(g\right)\\ {\mathrm{CO}}_{2}\left(g\right)⇌{\mathrm{CO}}_{2}(\mathrm{aq})\\ {\mathrm{CO}}_{2}\left(\mathrm{aq}\right)+H_{2}O\left(\mathrm{aq}\right)⇌{\mathrm{HCO}}_{3}^{-}+H^{+}\\ {\mathrm{HCO}}_{3}^{-}⇌H^{+}+{\mathrm{CO}}_{3}^{2-}\\ {\mathrm{Ca}}^{2+}+{\mathrm{CO}}_{3}^{2-}={\mathrm{CaCO}}_{3}\left(s\right) \end{array}$$

The morphology of sphere-shaped nanoparticles was confirmed by TEM and SEM (Additional file 1: Figure S1). As shown in Figure 2, nanoparticles synthesized via binary solvent method exhibited uniform size and good dispersal. It can be observed that the ECCNSs are large spheres with the diameter of about 2 μm. Meanwhile, some small nanocrystals with the size of about 50 to 200 nm (secondary nanoparticles) can also be observed in the PCS images (Additional file 2: Figure S2), which were possibly due to the decomposition of ECCNSs into the secondary nanoparticles when the pH decreased.

**Figure 3:**
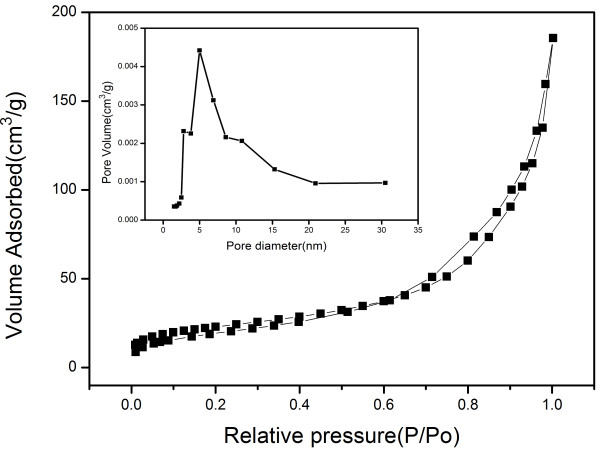
**Nitrogen adsorption-desorption isotherms of the obtained CCNSs.** Inset: Corresponding Barret-Joyner-Halender (BJH) pore size distribution curve determined from the N_2_ desorption isotherm.

The porous properties of CaCO_3_ products have been investigated by the N_2_ adsorption-desorption analyses (Figure 3). The obtained CaCO_3_ product has a high BET surface area of 82.14 m^2^/g, and the average pore size is 13.98 nm with narrow pore size distribution. Its BET specific surface is higher than that of the reported CaCO_3_[39,42].

**Figure 4:**
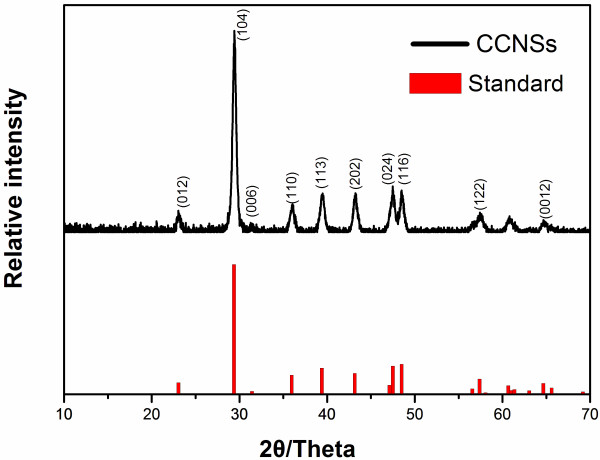
**X-ray diffraction patterns of CCNSs and the standard pattern of CaCO**_**3 **_**(JCPDS 47–1743).**

Figure 4 shows X-ray diffraction patterns of CCNSs prepared in the system of the binary solvent and the standard data of calcite (JCPDF-47-1743) as reference. By comparison with standard data of calcite, it was found that diffraction peaks of CCNSs were broadened due to the nanosize effect, and no peaks of other phases was found, indicating the CCNSs are well crystallized and of high purity. From the results of XRD, it can be seen that the samples retain the same crystal form of calcite.

CaCO_3_ shows two characteristic absorption peaks centered at 875 cm^−1^ (bending vibration of calcite) and 745 cm^−1^ (bending vibration of vaterite) in its infrared absorption spectrum [43]. In curve b of Additional file 3: Figure S3, CCNSs display three strong absorption bands at 875, 1426, and 712 cm^−1^, which are characteristic absorption bands of calcite. It indicated that CCNSs are the crystal form of calcite, which agrees with the results from XRD patterns. The spectra of etoposide (c) shows the following bands: 2,923 cm^−1^ (C-H stretch), 1,770 cm^−1^ (C=O stretch of ester bond), 1,613 cm^−1^ (C=O stretch of carboxyl methyl), and 1,056 cm^−1^ (C-O-C stretch), as well as the bands at 1,487 cm^−1^ and 1,405 cm^−1^, corresponding to the C=C stretching in the backbone of the aromatic phenylring. Compared with that of CCNSs (b) and etoposide (c), the spectra of ECCNSs (a) not only display the visibly characteristic bands of CaCO_3_ (with a small shift) but also show almost all etoposide characteristic vibration, which indicates that that etoposide was successfully packed into CCNSs.

**Figure 5:**
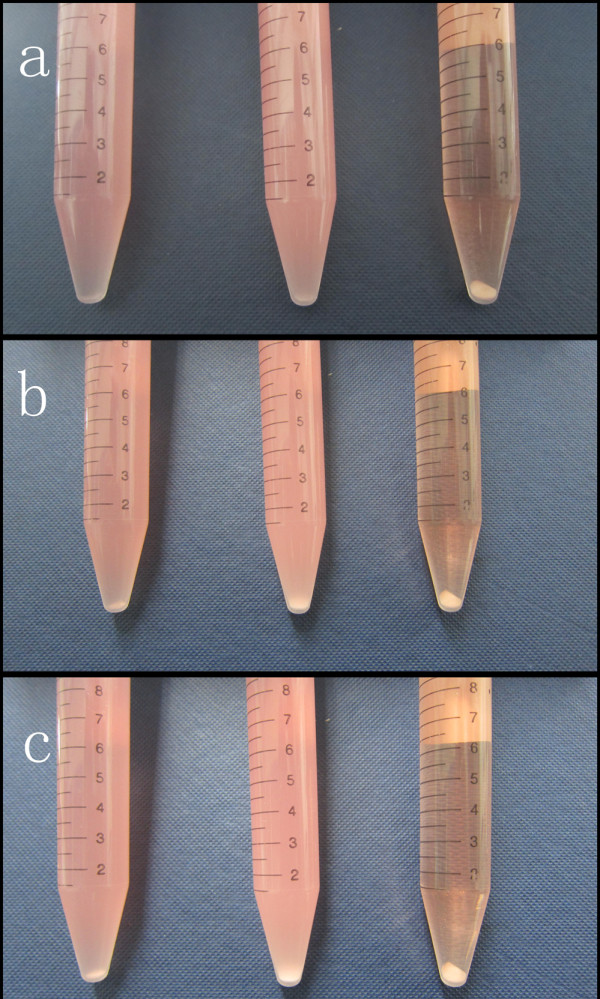
**Sedimentation photographs of CCNSs, ECCNSs, and free etoposide in RPMI-1640 medium.** After standing for 10 min **(a)**, 1 h **(b)**, and 2 h **(c)**.

Figure 5 presents the photographs of CCNSs, ECCNSs, and free etoposide in RPMI-1640 medium supplemented with 10% fetal bovine serum and 1% penicillin-streptomycin solution, which were recorded at 10 min, 1 h, and 2 h after standing. It can be seen that both CCNSs and ECCNSs disperse stably in RPMI-1640 medium, and little sedimentation of the particles was observed after standing for 2 h. In contrast with CCNSs and ECCNSs, the free etoposide added in RPMI-1640 medium began to precipitate and aggregate in the initial 10 min, and most part of the sample still precipitated at the bottom of the tube after standing for 2 h. Therefore, the embedding of etoposide into CCNSs obviously enhanced the dispersion and stability of the drug in medium solution.

**Figure 6:**
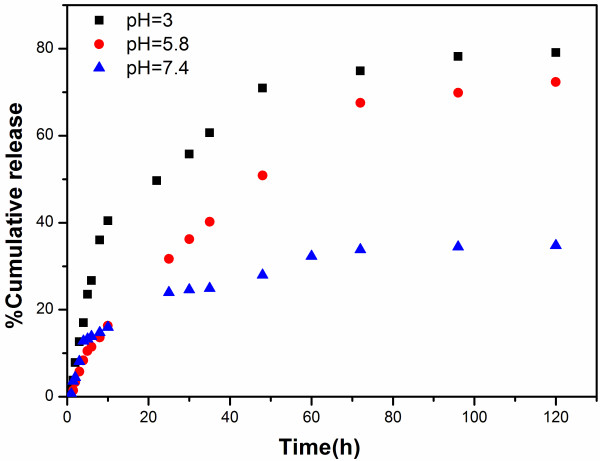
**Release profiles of etoposide from ECCNSs under simulated physiological conditions (pH 3.0, 5.8, and 7.4 at 37°C).**

The release of etoposide from ECCNSs *in vitro* is shown in Figure 6. The drug release behaviors of ECCNSs were studied at pH 7.4, 5.8, and 3, which modeled the different environments of blood and normal tissue (pH 7.4), tumor microenvironment (pH 5.8), and gastric juice (pH 3.0), respectively. The etoposide released from the highly ordered hierarchical calcium carbonate nanospheres gradually increase as the pH value decrease. There is an initial burst which could be attributed to the physical adsorption of a small amount of etoposide. Then, a sustained release from ECCNSs could be observed, and the cumulative drug release is about 80% at pH 3 after 120 h. At pH 7.4, the release amount was quite low and only approximately 30% was released in 120 h, which suggested that the delivery process might be governed mainly by diffusion from the outer drugs rather than the degradation of ECCNSs. At pH 5.8, about 50% of the loaded drug was released within 48 h, which was much lower than the drug release at pH 3. These results can demonstrate that the release of etoposide from ECCNSs is a pH-sensitive controlled release system, which is of particular feasibility in achieving the tumor-targeted therapy. Suppose that oral administration is chosen, the ECCNSs can ensure a stable delivery of etoposide during blood circulation. When the nanohybrids accumulate at the tumor site through the EPR effect, a fast and stable etoposide release can be triggered in response to extracellular or intracellular stimulus of tumor cells, where pH value is lower than that in the normal tissue.

**Figure 7:**
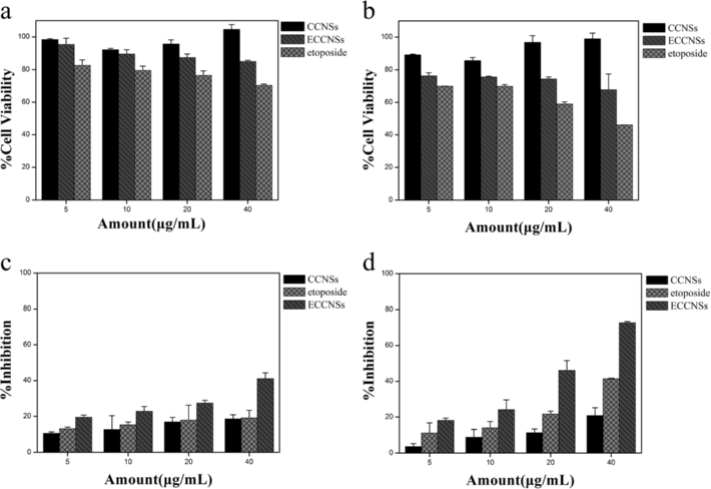
**The viability of HEK 293 T and SGC-7901 cells influenced by CCNSs, free etoposide, and ECCNSs. (a)** and **(b)** growth inhibition assay results for HEK 293 T cell line with CCNSs, free etoposide, and ECCNSs after 24 and 48 h incubation. Diagrams were plotted as particle concentrations of 5, 10, 20, and 40 μg/mL. **(c)** and **(d)** growth inhibition assay results for SGC-7901 cell line with CCNSs, free etoposide, and ECCNSs after 24 and 48 h incubation. Diagrams were plotted as etoposide concentrations of 5, 10, 20, and 40 μg/mL. All experiments were carried out in triplicate.

Figure 7a, b shows the viability of normal cells (HEK 293 T) treated with CCNSs, free etoposide, and ECCNSs at various concentrations (5, 10, 20, 40 μg/mL), for 24 and 48 h. HEK 293 T cells treated with CCNSs all show over 80% survival rate, which indicates that the CCNSs show low cytotoxicity and have good biocompatibility. Compared with free etoposide, ECCNSs showed obviously lower cytotoxicity against normal cells. It can be inferred that embedding of etoposide into CCNSs can alleviate the cytotoxicity of etoposide to normal cells.

Figure 7c, d shows the effect of etoposide formulation on the inhibition against SGC-7901 cell growth. The results showed the suppression of SGC-7901 cell growth by different nanohybrids was concentration and time dependent. The inhibition rates of ECCNSs and the free etoposide are 72.66% and 41.40% over 48 h, respectively. Obviously, ECCNSs showed higher suppression efficiency than free etoposide against the growth of SGC-7901 cells. Synergistic therapeutic effects occurred when etoposide was entrapped by CCNSs. It is possible that good dispersivity and stability of ECCNSs in culture medium (Figure 5) may lead to a greater cellular uptake than that of free etoposide.

Then, the pH values of culture media for SGC-7901 cells were measured as 8.1 (0 h), 7.82 (24 h), and 6.76 (48 h). Therefore, it can be inferred that the release of etoposide from ECCNSs may increase as the pH value of the culture decreases because of its pH-sensitive controlled release behavior investigated above. The stronger cell inhibition of ECCNSs further confirms that the cell uptake of nanoparticles, the decomposition of ECCNSs as the pH descends, and the passive diffusion of the free etoposide released from the ECCNSs, together helped to achieve the cell inhibition effect.

**Figure 8:**
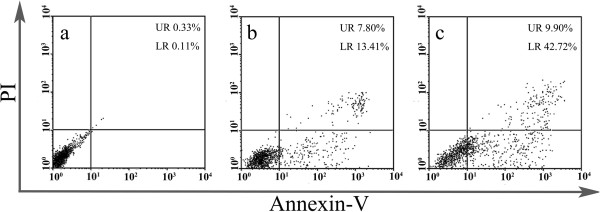
**FACS analysis of SGC-7901 cells stained with Annexin V-FITC and PI. (a)** Cells did not treat with any agents as blank control, **(b)** cells apoptosis induced by VP-16, **(c)** cells treated with the ECCNSs. In all panels, LR represents early apoptosis and UR represents late apoptosis.

The mechanism of cell growth inhibition by ECCNS nanoparticles was studied using Annexin V-FITC Apoptosis Detection Kit. As we know, early apoptosis was characterized by plasma membrane reorganization and was detected by positive staining for Annexin V-FITC while later stage apoptosis was characterized by DNA damage and detected by positive staining for both Annexin V and PI. In this study, we stained SGC-7091 cells with Annexin V-FITC and PI after the treatment of free etoposide or ECCNSs (30 μg/mL) nanoparticles for 24 h. Meanwhile, cells without any addition were set as control. As given in Figure 8a, SGC-7901 cells without any additive showed 0.11% early apoptosis and 0.33% later apoptosis. The treatment with etoposide led to 13.41% early apoptosis and 7.80% later apoptosis (Figure 8b). The results clearly reveal that the early apoptosis increased to 42.72% and later apoptosis increased to 9.90% (Figure 8c) when the cells were treated with ECCNSs. It is now well established that etoposide-induced cleavage of DNA by topoisomerase II can mediate the formation of chromosomal translocation breakpoints, leading to the expression of oncogenic factors responsible [44]. Etoposide can cause apoptosis cascade in gastric cancer cells by coupling DNA damage to p53 phosphorylation through the action of DNA-dependent protein kinase [45]. The percentage of both early apoptosis and later apoptosis in the ECCNSs-treated group remarkably increased compared with free etoposide alone and untreated control, which indicated that ECCNSs were able to accelerate the apoptosis processes of tumor cells. The result also revealed that etoposide entrapped in CCNSs could enhance the efficient antitumor effect.

**Figure 9:**
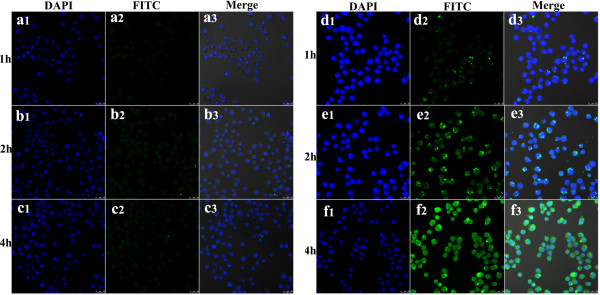
**Confocal laser scanning microscopy images of the etoposide. (**Rows **a**, **b**, and **c)** and ECCNSs **(**rows **d**, **e**, **f)** on SGC-7901 cells. At the predetermined point of 1, 2, and 4 h. In each case, 1, 2, and 3 indicate DAPI, FITC, and Merge, respectively. The scale bar represents 25 μm.

The CLSM image of the etoposide/ECCNSs is shown in Figure 9. The high therapeutic effect by ECCNSs was investigated by the uptake behavior in SGC-7901 cells. Thus, the effective therapy may result from the enhanced intracellular delivery, the pH-sensitive release, and protection of etoposide by ECCNSs. Etoposide (rows a, b, c) and ECCNSs (rows d, e, f) passed through the cell membrane of SGC-7901 cells and assembled in nucleus at the predetermined point of 1, 2, and 4 h. These results demonstrated that cellular uptake of SGC-7901 cell was time-dependent, and the efficient cellular uptake of ECCNSs was higher than that of the free etoposide. From the CLSM image, it could also be seen that the CCNS carriers could aggregate around the nucleus (blue fluorescence) and even directly intrude into the nucleus.

**Figure 10:**
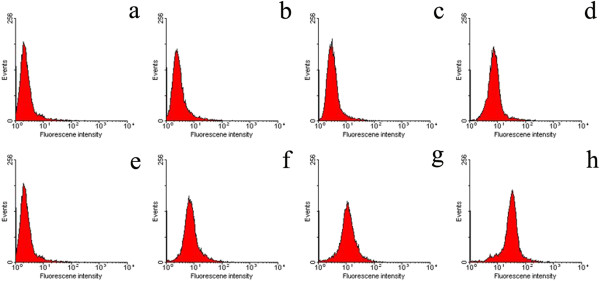
**SGC-7901 cells were treated with 30 μg/mL etoposide in two forms of ECCNSs (f, g, and h) and void etoposide (b, c, and d).** As the plots show, the number of events (*y*-axis) with high fluorescence intensity (*x*-axis) increases by 4-h incubation with ECCNSs but without any evident change for void etoposide. Negative control **(a** and **e)** includes nontreated cells to set their auto-fluorescence as ‘0’ value.

Kinetic assessment of ECCNSs (Figure 10b, c, d) uptake and void etoposide (Figure 10f, g, h) in SGC-7901 cell was conducted by plotting the fluorescence peak of each sample against the different incubation times of 1 h (b, f), 2 h (c, g), and 4 h (d, h). The number of events with high intensity for 30 μg/mL etoposide increased when the incubation time continued to 4 h, pretending its uptake into cells. At the same time, etoposide did not show any significant change in fluorescence intensity compared with ECCNSs. As shown in Figure 10, the cellular fluorescence is much higher when the etoposide was loaded on CCNSs, which indicates that CCNSs, as a drug carrier, enhances the cell uptake of etoposide. The fluorescence intensity of the ECCNSs and etoposide is in agreement with the results from CLSM images.

Controlled delivery of drug using carrier materials is based on two strategies: active and passive targeting. The former is technical sophisticated and suffering from many difficulties. Otherwise, the latter is easier to implement practically [46]. Many formulations have been used in the representative passive-targeting strategies based on the EPR effect [47]. Tumor vessels are often dilated and fenestrated due to rapid formation of vessels that can serve the fast-growing tumor while normal tissues contain capillaries with tight junctions that are less permeable to nanosized particle [11,48]. The EPR effect is that macromolecules can accumulate in the tumor at concentrations five to ten times higher than in normal tissue within 1 to 2 days [49]. Besides, biomaterials with diameters more than 100 nm tend to migrate toward the cancer vessel walls [50]. Therefore, the EPR effect enables ECCNSs (secondary nanoparticles) to permeate the tumor vasculature through the leaky endothelial tissue and then accumulate in solid tumors. On one hand, the uptake of ECCNSs by tumor cells can lead to the direct release of etoposide into intracellular environment to kill tumor cells. On the other hand, the pH-sensitive drug release behavior for ECCNSs may lead to the low release of etoposide from ECCNSs in pH neutral blood, and the rapid release of the drug in relatively acidic extracellular fluids in the tumor. In this way, the targeted delivery of etoposide to tumor tissues may be possible by ECCNSs.

**Figure 11:**
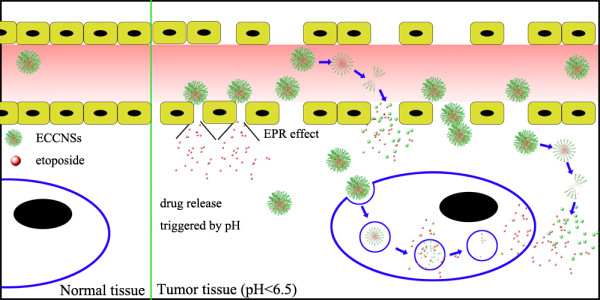
A representative illustration of ECCNSs targeting.

Referring to some previous reports [51,52], the possible mechanism for the targeted delivery of the ECCNSs is illustrated in Figure 11. Most of the biodegradable ECCNSs decompose into the secondary nanoparticles in the vicinity of the tumor endothelium, with the release of epotoside. The small therapeutic nanoparticles and drugs readily pass through the endothelia into tumor tissues for efficient permeability [53]. The degradation of the materials in the endosomes or lysosomes of tumor cells may determine the almost exclusive internalization along clathrin-coated pits pathway. The multistage decomposition of ECCNSs in blood vessels or tumor tissue is likely to play a key role in determining their targeting and biological activity [54].

## Conclusions

In summary, we have proposed a facile method to prepare mesoporous CCNSs formed by the multistage self-assembled strategy in a binary solvent reaction system. Further studies on CCNSs as carriers for etoposide (loading capacity 39.7%) demonstrated their pH-sensitive drug release profile and enhanced cytotoxicity by increasing cellular uptake and apoptosis to tumor cell. The cytotoxicity test and apoptosis test showed that the carrier of CCNSs was almost nontoxic and ECCNSs were evidently more efficient than free etoposide in antitumor effect and deliver activity. These results also indicated that the hierarchical mesoporous CaCO_3_ nanospheres (CCNSs) hold great promise to overcome the drawbacks of water-insoluble drugs such as etoposide and thereby enhance their therapeutic effect.

## Abbreviations

CCNSs: Calcium carbonate nanospheres; CLSM: Confocal laser scanning microscopy; ECCNSs: Etoposide-loaded CCNSs; EPR effect: Enhanced permeation and retention effect; FACS: Fluorescence-activated cell sorter; SEM: Scanning electron microscope; TEM: Transmission electron micrograph.

## Competing interests

The authors declare that they have no competing interests.

## Authors' contributions

HP carried out the cell studies (MTT assay and CLSM test) and drafted the manuscript. KL carried out the preparation of nanoparticles. TW carried out the apoptosis test studies. JW carried out the *in vitro* drug release studies. JW carried out the characterization of nanoparticles. RZ carried out the sedimentation study. DS participated in the design of the study and performed the statistical analysis. SW conceived of the study and participated in its design and coordination. All authors read and approved the final manuscript.

## Authors' information

DS and RZ are assistant professors. SW is a professor, and HP, KL, TW, JW, and JW are graduate students from the School of Life Science and Technology, Tongji University.

## Supplementary Material

Additional file 1: Figure S1 TEM and SEM images of a series of intermediates trapped during the reaction. Click here for file

Additional file 2: Figure S2 Particle size distributions of CCNSs (a) and ECCSs (b). Click here for file

Additional file 3: Figure S3 FT-IR spectra of (curve a) ECCNSs (curve b) CCNSs, and (curve c) etoposide. Click here for file
