# Supplementary figures and images for: Preparation of hierarchical mesoporous CaCO3 by a facile binary solvent approach as anticancer drug carrier for etoposide
Source: Nanoscale Res Lett. 2013 Jul 15;8(1):321. doi: 10.1186/1556-276X-8-321 (PMC3716939; doi:10.1186/1556-276X-8-321)

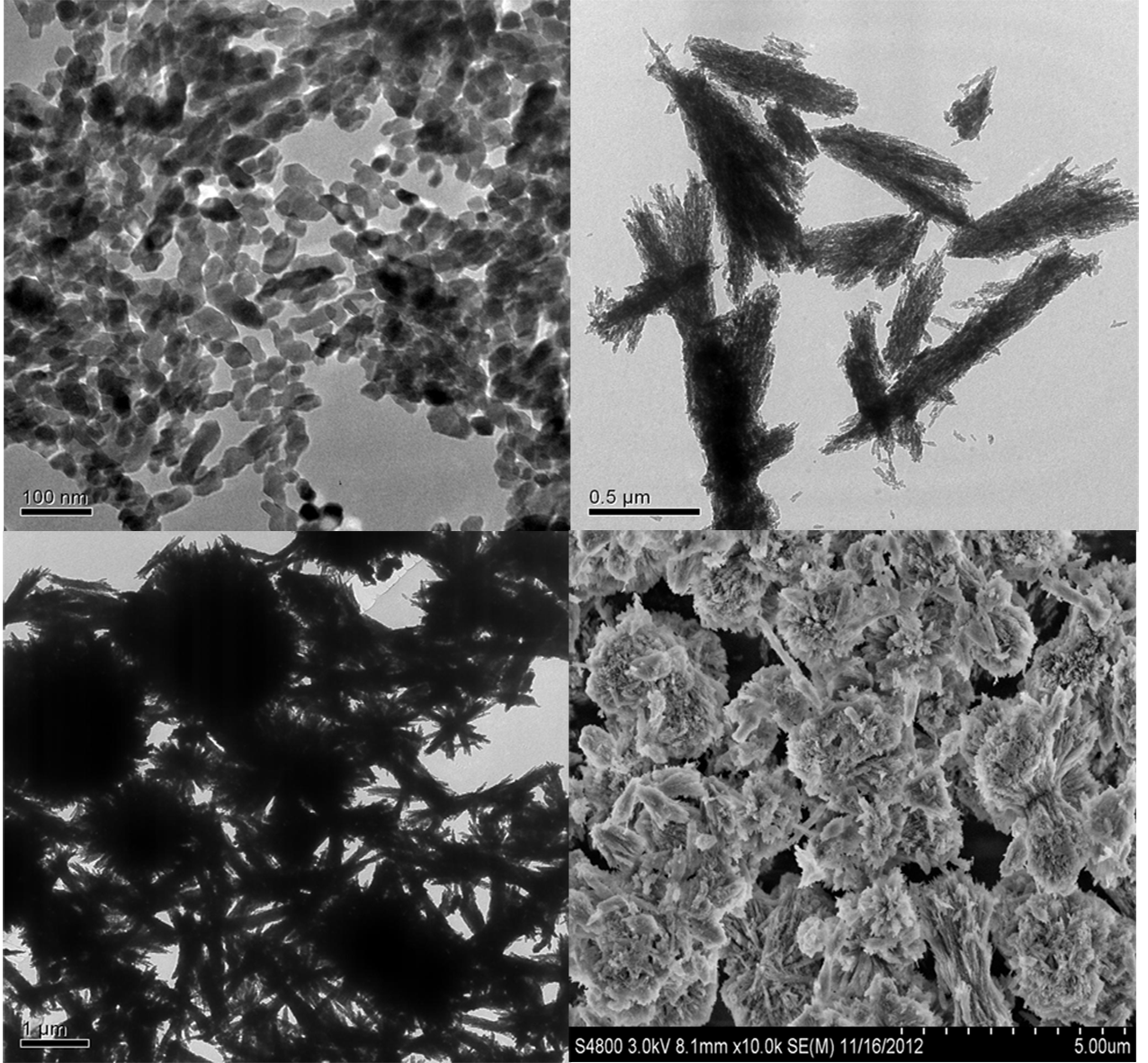

Supplement: Additional file 1: Figure S1 — TEM and SEM images of a series of intermediates trapped during the reaction. [file 1556-276X-8-321-S1.tiff]

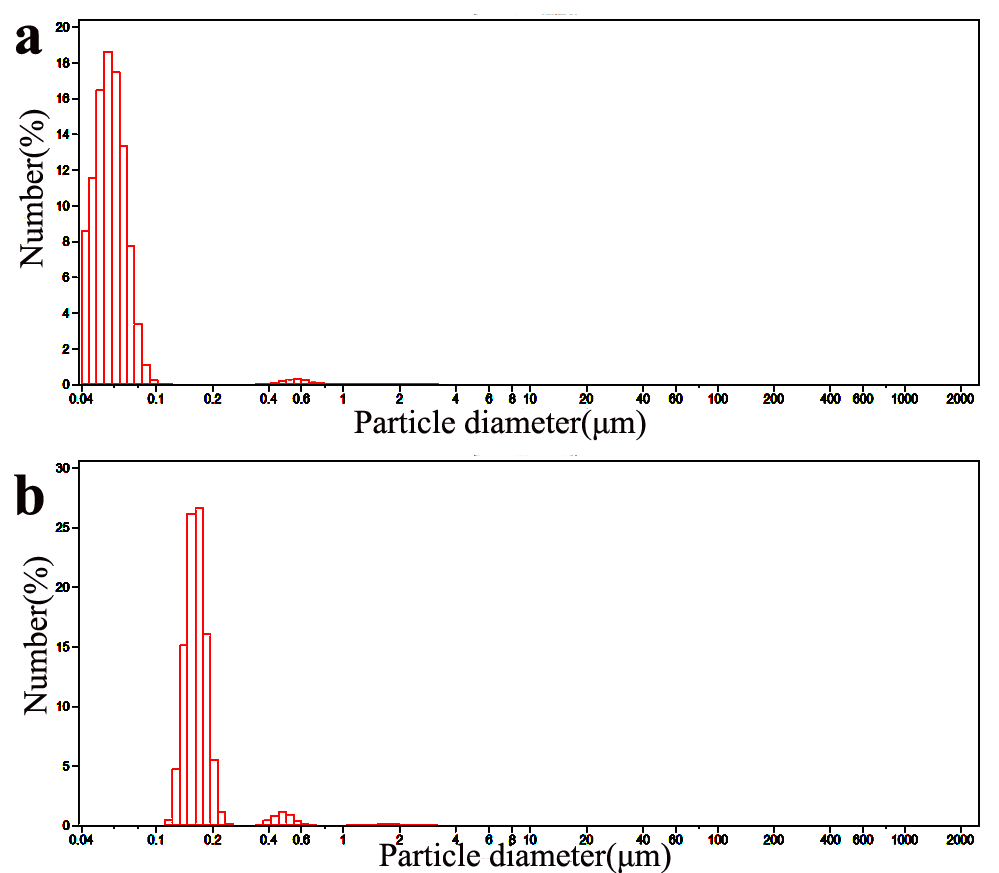

Supplement: Additional file 2: Figure S2 — Particle size distributions of CCNSs (a) and ECCSs (b). [file 1556-276X-8-321-S2.tiff]

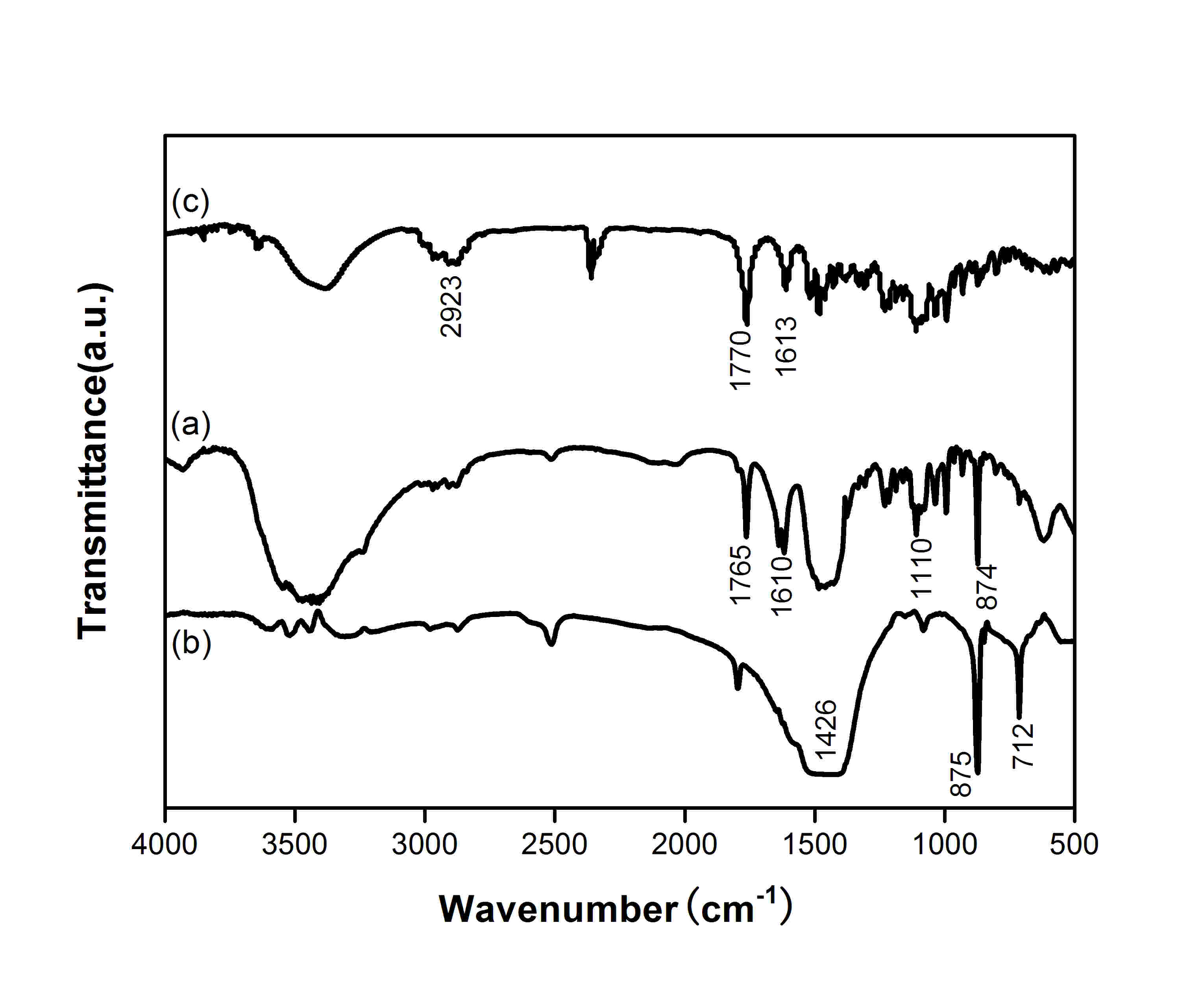

Supplement: Additional file 3: Figure S3 — FT-IR spectra of (curve a) ECCNSs (curve b) CCNSs, and (curve c) etoposide. [file 1556-276X-8-321-S3.jpg]
